# Significant decrease in peripheral regulatory B cells is an immunopathogenic feature of dermatomyositis

**DOI:** 10.1038/srep27479

**Published:** 2016-06-07

**Authors:** Wenli Li, Xiaolan Tian, Xin Lu, Qinglin Peng, Xiaoming Shu, Hanbo Yang, Yuanli Li, Yan Wang, Xuezhi Zhang, Qingyan Liu, Guochun Wang

**Affiliations:** 1 Department of Rheumatology, China-Japan Friendship Hospital, Beijing, People’s Republic of China

## Abstract

Regulatory B cells (Bregs) are critical in maintaining self-tolerance. Their role in dermatomyositis (DM), an autoimmune disease characterized by inappropriate regulation of hyperactivated B and T cells, has not been clearly defined. In the current study, we performed flow cytometry analysis of studied CD19^+^ CD24^high^CD38^high^ Breg subpopulations in blood samples from 30 patients with DM, 37 diseased controls and 23 healthy controls. A significant decrease was observed in the frequency of Bregs in DM patients compared to that in diseased controls (*p* < 0.0001) and in healthy controls (*p* < 0.0001). And the prevalence of Bregs deficiency (defined as Bregs/B cells < 0.50% in this study) in DM patients went as high as 73.3%. Furthermore, DM patients with positive myositis specific autoantibody often had lower Bregs levels than negative patients (*p* = 0.036), and lower level of Bregs was also found in DM patients with interstitial lung disease than in DM patients without (*p* = 0.041). In a follow-up study, seven DM patients were considered to be in remission stage, and their Breg levels were found to have significantly increased after treatment (*p* = 0.022). Our research revealed that Breg deficiency is an immunopathogenic feature of DM and provided insights into the design of new immunotherapy target for DM clinical interventions.

Dermatomyositis (DM) is a systemic autoimmune disorder that is characterized by the inflammation of the muscles and the skin. It may also affect the joints, the esophagus, the lungs, and the heart^1^. Immunological dysfunctions have been described in patients with DM, including expanded autoaggressive T cells and B cells, aggregates of lymphocytes in skin lesions, increased autoantibody production, and aberrant cytokine production^2^,^3^. Although understanding the pathophysiology of DM remains a great elusive challenge, a thorough examination of self-tolerance maintenance may provide an important clue to understanding the pathogenesis of the disease.

Recently, regulatory B cells (Bregs) have been identified as a key regulator and show a potent immunosuppressive activity^4^,^5^. These immunoregulatory B cells are capable of suppressing auto-reactive lymphocytes that escape from central tolerance mainly through producing of the anti-inflammatory cytokines, such as interleukin-10 (IL-10)^6^,^7^,^8^. In mice, Bregs have been found to help maintain immunologic homeostasis in tissues and play a role in preventing autoimmune induction, and adoptive transfers of Bregs confirmed the role of these cells in attenuating excessive autoimmune inflammation^9^,^10^.

In humans, although there was no unique marker or set of markers that could exclusively identify the Bregs, the majority of human IL-10^+^ regulatory B cells are enriched within CD19^+^ CD24^high^CD38^high^ B cells subset, which have similar tolerance-inducing role as Bregs in mice^11^,^12^. During the last five years, Bregs have been investigated in several autoimmune diseases, such as systemic lupus erythematosus (SLE), rheumatoid arthritis (RA), primary Sjögren’s syndrome (pSS), pemphigus, and so on^13^,^14^,^15^,^16^. The growing mass of evidence has suggested that numerical or functional modifications of Breg cells could lead to the breakdown of self-tolerance and the emergence of autoimmunity. However, results have been inconclusive because decreased, normal, and even increased levels of Bregs have been observed in different kinds of autoimmune diseases.

To date there is no data about the role of Bregs in the pathogenesis of DM, and the number and significance of Bregs in DM remains unclear. Given the fact that both cellular and humoral immunity have been implicated in the pathogenesis of DM, and considering the key role that Bregs play in immunomodulation, it is reasonable to speculate that Bregs may play a pivotal role in the pathomechanism of DM. Our study sought to investigate the presence and phenotypic characteristics of CD19^+^ CD24^high^CD38^high^ B lymphocytes in the peripheral blood of patients with DM, and their implication in the extent of the disease activity.

## Results

### Breg cell numbers are remarkably reduced in patients with DM

**Figure 1:**
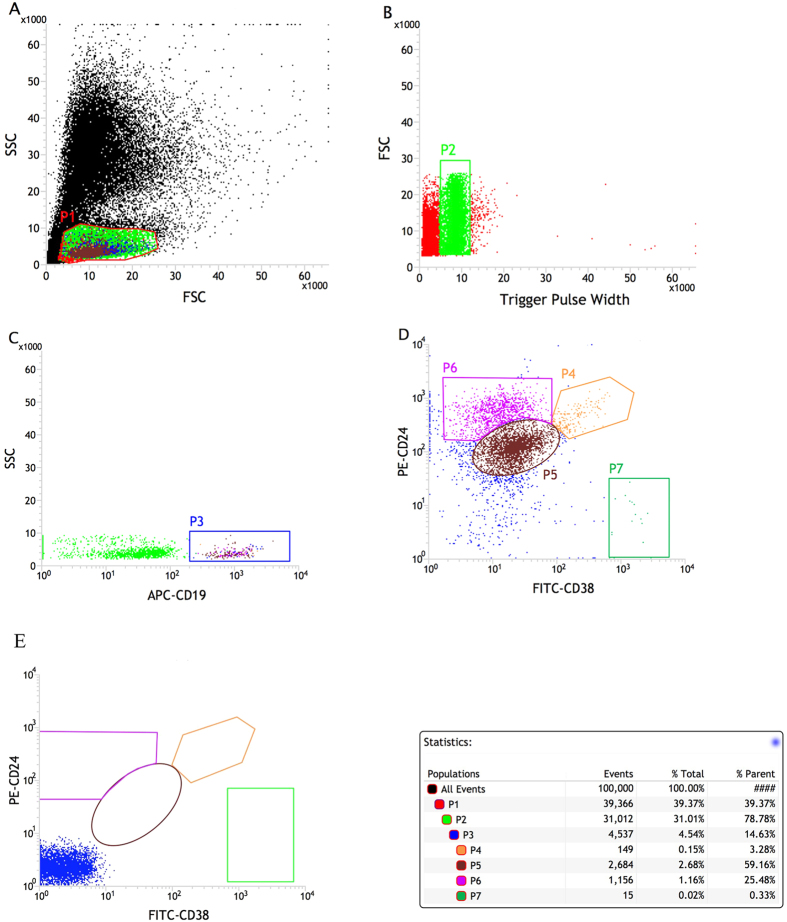
Multi-parameter strategy for analysis to identify Bregs. A representative healthy control sample was analyzed by flow cytometry according to the expression of CD19, CD24, and CD38. (**A**) The lymphocyte population was gated according to forward- and side-scattered properties(P1); (**B**) Doublets were removed according to the trigger pulse width(P2); (**C**) CD19^+^ B cells were gated using anti-CD19 APC antibodies(P3). (**C**) According to the intensity of CD24 and CD38 expression, CD19^+^ B cells were subdivided into four populations: CD19^+^ CD24^high^CD38^high^ (P4), CD19^+^ CD24^intermediate^CD38^intermediate^ (P5), CD19^+^ CD24^high^CD38^low^ (P6) and CD19^+^ CD24^low^CD38^high^ (P7). (**E**) The corresponding isotype control.

Peripheral blood lymphocytes from 30 patients with DM, 37 patients with other autoimmune diseases (15 cases of SLE, 15 cases of RA and 7 cases of pSS), and 23 healthy individuals were phenotypically analyzed by flow cytometry for their expression of CD19, CD24, and CD38 surface markers. The percentages of cell subset populations were calculated as the ratios of gated cells to total B cells and isotype controls were used to set up the negative population. The results were expressed as mean values ± SEM. The gate strategy for Bregs (CD19^+^ CD24^high^CD38^high^) was illustrated by a representative staining of Bregs in a healthy control subject (Fig. 1).

**Figure 2:**
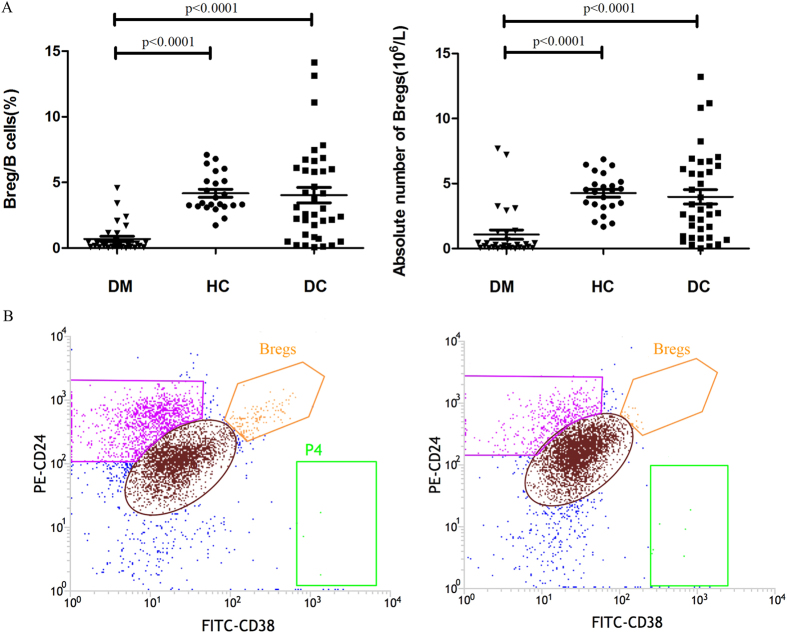
(**A**) The Breg levels among DM patients (n = 30), diseased controls (DC) (n = 37), and healthy controls (HC) (n = 23). The results were expressed by scatter dots plot depicting mean ± SEM. A significant decrease was observed in the frequency of CD19^+^ CD24^high^CD38^high^ Bregs in DM patients compared to those in healthy controls (0.713 ± 0.275% vs 4.196 ± 0.347%, *p* < 0.0001) and in diseased controls (0.713 ± 0.275% vs 4.010 ± 0.566%, *p* < 0.0001) (**A**, left). The absolute number of Bregs was also significantly lower in DM patients than in healthy controls (1.079 ± 0.358 × 10^6^ vs 4.267 ± 0.301 × 10^6^, *p* < 0.0001) and diseased controls (1.079 ± 0.358 × 10^6^ vs 3.981 ± 0.551 × 10^6^, *p* < 0.0001) (**A**, right). (**B**) Representative cases of a healthy control (**B**, left) and of a DM patient (**B**, right).

Bregs levels in DM patients, diseased controls, and healthy controls are shown in Fig. 2A. We observed a significant decrease in the levels of CD19^+^ CD24^high^CD38^high^ Bregs in DM patients compared to those in healthy controls (0.713 ± 0.275% vs 4.196 ± 0.347%, p < 0.0001) and to those in diseased controls (0.713 ± 0.275% vs 4.010 ± 0.566%, p < 0.0001). The absolute number of Bregs was also significantly lower in DM patients than in healthy controls (1.079 ± 0.358 × 10^6^ vs 4.267 ± 0.301 × 10^6^, *p* < 0.0001) and diseased controls (1.079 ± 0.358 × 10^6^ vs 3.981 ± 0.551 × 10^6^, *p* < 0.0001) There was no significant difference between the diseased controls sub-groups regarding their Bregs percentages (RA vs SLE vs pSS: 3.450 ± 0.613% vs 4.753 ± 0.472% vs 4.434 ± 1.109%, *p* > 0.05). It is noteworthy that Bregs population was almost absent in quite a number of DM patients, if we defined the Bregs deficiency as Bregs/B cells < 0.50% in our study, the prevalence of Bregs deficiency among DM patients could reach 73.3% (22/30). Representative cases of a DM patient and of a healthy control are shown in Fig. 2B.

In addition to the CD19^+^ CD24^high^CD38^high^, two other distinct B cell populations can be observed in the flow cytometry plot: CD19^+^ CD38^int^CD24^int^ B cells (primarily mature B cells), and CD19^+^ CD24^high^CD38^−^ B cells (primarily memory B cells)^17^,^18^. In the research, we observed significantly higher percentages of CD19^+^ CD38^intermediate^CD24^intermediate^ B cells in patients with DM compared to those in healthy individuals (66.98 ± 2.321% vs 58.25 ± 1.836%, *p* < 0.001). In contrast, the percentages of CD19^+^ CD24^high^CD38-B cells in patients with DM and those in healthy controls were not significantly different (21.33 ± 2.456% vs 23.57 ± 1.858%, *p* > 0.05).

Of the 30 DM patients in our cohort, 7 patients were newly diagnosed and treatment naïve. Therapeutic strategies for all the patients have been presented in Supplementary Table S1 and Table 1. We did not find a significant difference in Bregs levels between treatment naïve DM patients and DM patients received treatment (0.692 ± 0.22% vs 0.727 ± 0.538%, p > 0.05).

**Figure 3:**
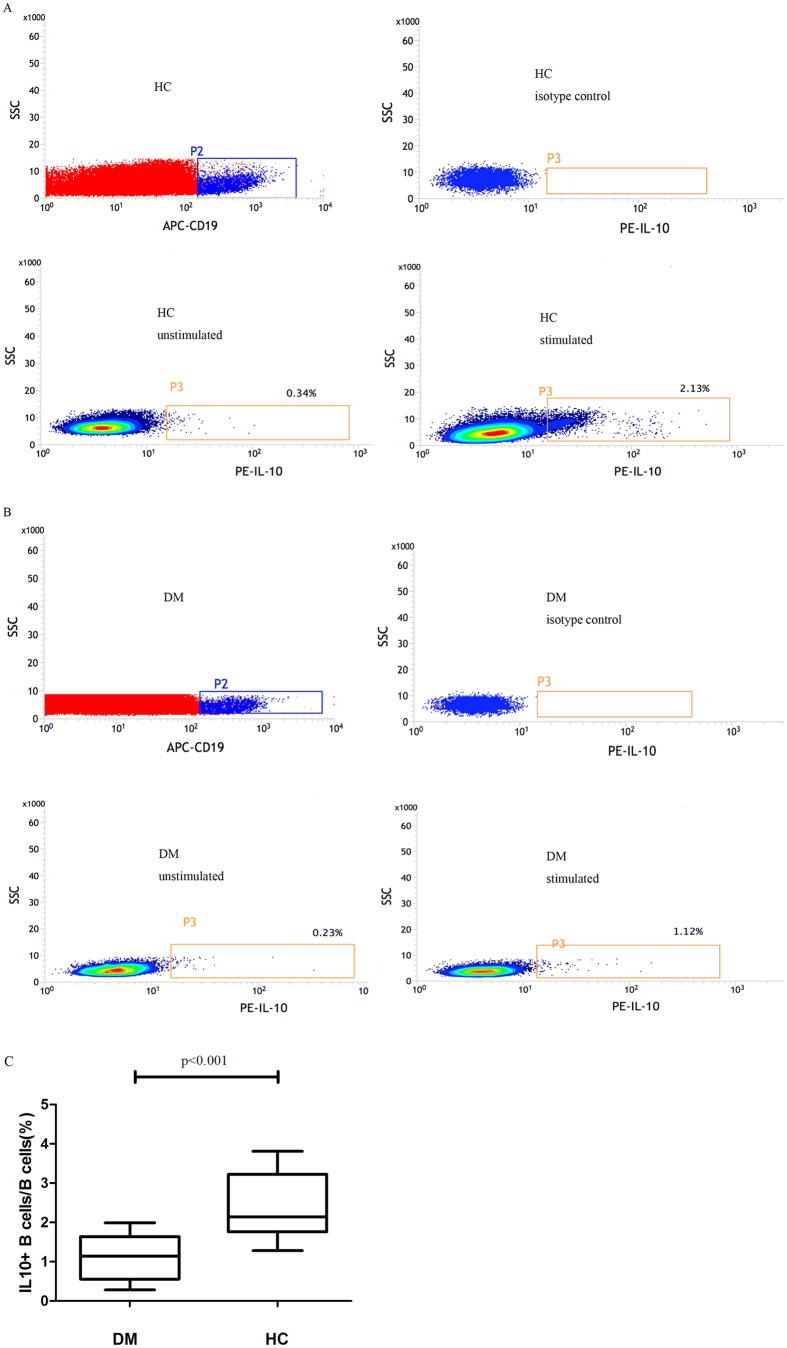
Analysis of intracellular IL-10 production. (**A,B**) Representative B cell cytoplasmic IL-10 expression in healthy individuals (**A**) and in DM patients (**B**) after 6 hours of *in vitro* stimulation with Leukocyte Activation Cocktail, surface staining with APC-labeled anti-CD19, and intracellular staining with PE-labeled anti–IL-10. (**C**) the frequency of IL-10^+^ B cells was significantly lower in DM patients than in healthy controls (1.139 ± 0.156% vs 2.372 ± 0.205%, *p* < 0.001).

Similarly, percentages of B cells with positive intracellular IL-10 (IL-10^+^ B cells) were significantly lower in DM patients than in healthy controls (1.139 ± 0.156% vs 2.372 ± 0.205%, *p* < 0.001). Representative B cell cytoplasmic IL-10 expressions by DM patients and by healthy controls are shown in Fig. 3.

### CD19 expression in B cells is decreased in DM patients

**Figure 4:**
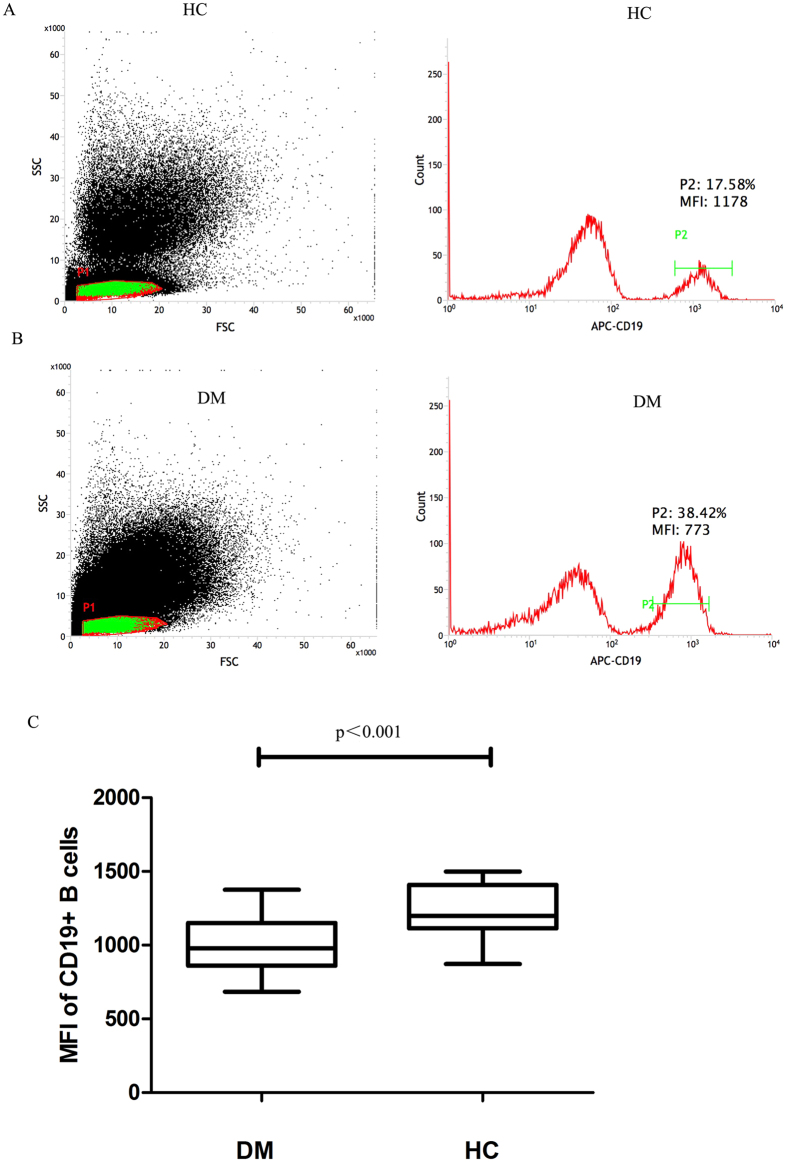
CD19 expression in B cells. Comparing CD19 MFI of B cells between patients with DM and HCs. (**A,B**) In these representative cases, obviously, the mean percentages of total B cell is higher in the DM patient than in the healthy individual (38.42% vs 17.58%). CD19 expression is lower among all the B cells of the DM patient compared to that of the healthy control (MFI: 773 vs 1178). (**C**) The mean CD19 MFI expression of B cells was significantly decreased in the DM group compared to the healthy control group (mean MFI: 999 ± 40.69 vs 1222 ± 42.75, *p* < 0.001).

Because CD19 molecules are important for Breg cell proliferation and survival, we assessed CD19 expression in the B cells of DM patients and in those of healthy individuals. Levels of CD19 expression were calculated as geometric mean fluorescence intensity (MFI). Briefly, the lymphocyte population was gated according to forward- and side-scattered properties, B cells were gated using anti-CD19 APC antibodies. The sub-gated B cells were then assessed by MFI analysis to measure their level of CD19 expression. In these representative cases (Fig. 4), it is clear that CD19 expression is lower in DM patients than in healthy individuals. The mean level of CD19 expression (expressed as MFI) in B cells was 999 ± 40.69 in DM patients compared to 1222 ± 42.75 in HCs (*p* < 0.001).

### Reduced CD19^+^ CD24^high^CD38^high^ Breg levels are associated with myositis-specific autoantibodies production in patients with DM

**Figure 5:**
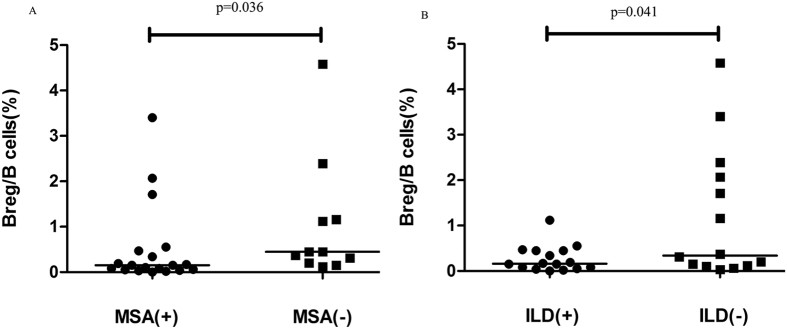
The frequencies of CD19^+^ CD24^high^CD38^high^ Bregs in DM patients with different clinical features. (**A**) Levels of CD19^+^ CD24^high^CD38^high^ Bregs were significantly lower in the MSA-positive group (n = 19) than in the MSA-negative group (n = 11) (*p* = 0.036); (**B**) A significantly lower percentage of CD19^+^ CD24^high^CD38^high^ Bregs was found in DM patients with interstitial lung disease (ILD) involvement (n = 16) than in the DM patients without ILD (n = 14) (*p* = 0.041).

Possible putative correlations between the percentages of CD19^+^ CD24^high^CD38^high^ Bregs and several laboratory parameters were examined in this study. There is no notable relationship between the percentages of CD19^+^ CD24^high^CD38^high^ Bregs and the levels of creatine kinase levels, erythrocyte sedimentation rate (ESR), C-reactive protein (CRP) levels, or plasma immunoglobulin A/G/M levels. However, a significantly lower percentages of CD19^+^ CD24^high^CD38^high^Bregs were found in the samples of myositis-specific autoantibody (MSA)-positive patients than in MSA-negative patients [0.15(0.02–3.42)% vs 0.43(0.12–4.60)%, *p* = 0.036] (Fig. 5A).

Of the 30 DM patients, there are 7 patients with anti-melanoma differentiation-associated gene 5 (MDA5) autoantibodies positive. Further analysis showed that DM patients with anti-MDA5 autoantibodies positive were prone to have significantly lower levels of Bregs compared to the anti-MDA5 antibody negative individuals (0.187 ± 0.06% vs 0.854 ± 0.253%, p = 0.017).

Bregs levels of ANA-positive group also displayed a trend toward being lower than those of the negative group [0.16(0.03–2.37)% vs 0.34(0.02–6.61)%, *p* > 0.05], although this difference was not statistically significant.

### Reduced CD19^+^ CD24^high^CD38^high^ Breg levels are associated with interstitial lung disease (ILD) involvement

We further examined the relationships between Bregs levels and the clinical manifestations. A significantly lower percentage of CD19^+^ CD24^high^CD38^high^ Bregs was found in DM patients with ILD than in DM patients without ILD [0.15(0.02–1.10)% vs 0.34(0.03–4.60)%, *p* = 0.041] (Fig. 5B). However, no significant difference were observed when Breg levels were compared in DM patients sub-grouped based the presence of amyosthenia and oropharyngeal dysphagia.

### CD19^+^ CD24^high^CD38^high^ Breg levels are associated with disease activity in a follow-up study

**Figure 6:**
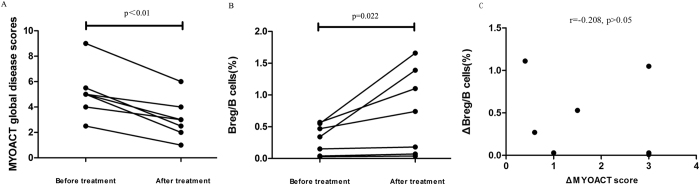
CD19^+^ CD24^high^CD38^high^ Bregs levels were analyzed in seven individual patients with DM before and after treatment. (**A**) After treatment, the MYOACT global disease scores of these seven patients were significantly lower (*p* < 0.01). (**B**) Breg precentages were significantly increased in DM patients after treatment compared to the percentages before treatment (*p* = 0.022). (**C**) There was no significant correlation between the changes in MYOACT score (ΔMYOACT) and the changes in Bregs level (ΔMYOACT) (r = −0.208, *p* > 0.05).

We then examined whether the frequencies of CD19^+^ CD24^high^CD38^high^ Bregs correlate with disease activity. We performed a mini follow-up study that analyzed seven patients with DM before and after treatment. After treatment, all seven patients were considered to be in remission, and their MYOACT global disease scores had significantly decreased (Fig. 6A, *p* < 0.01). Additionally, significantly increased Breg percentages were found in these DM patients after treatment compared with their percentages before treatment (Fig. 6B, *p* = 0.022), supporting the existence of an association between Breg level and disease activity. However, there was no correlation was found between the changes in MYOACT score (ΔMYOACT) and changes in Breg/B cells (ΔBregs) (r = −0.208, *p* > 0.05) (Fig. 6C).

### There is no notable relationship between Bregs levels and serum levels of serum IL-10, TNF-α, IFN-γ and IL-17

It has been reported that CD19^+^ CD24^high^CD38^high^ Bregs can suppress Th1 and Th17 differentiation as well as the release of TNF-α, IFN-γ and IL-17 by these cells, partially through the release of IL-10^14^. In our study, we measured multiple cytokines simultaneously and looked for any correlation they may have with Bregs levels. However, no notable relationship was observed between the percentages of CD19^+^ CD24^high^CD38^high^ Bregs and the serum levels of serum TNF-α, IFN-γ and IL-17. Furthermore, the levels of these cytokines in the DM patients and healthy controls were compared (Table 2). The TNF-α, IFN-γ and IL-17 levels were significantly higher in patients with DM than in healthy controls (*p* = 0.018, 0.006 and 0.020, respectively). However, no expected difference was observed in the level of IL-10 between the two groups (*p* > 0.05).

## Discussion

In this study, we identified and quantified the CD19^+^ CD24^high^CD38^high^ Bregs population in DM patients for the first time. We found that Bregs levels were remarkably decreased in DM patients compared to those in our two control populations. Bregs deficiency was also found to relate closely with DM clinical features and disease activity. DM patients with ILD often had lower Bregs levels than DM patients without ILD; and lower levels of CD19^+^ CD24^high^CD38^high^ Bregs were found in DM patients who were positive for MSA than in those who were MSA-negative. In our follow-up study, the Breg levels of DM patients after treatment were found to be significantly higher than they were before treatment, accompanied by decreased MYOACT global disease scores. Our research revealed that the decreased peripheral Bregs levels are an immunopathogenic feature of DM and provided insights into the design of new immunotherapy target for DM clinical interventions.

CD19^+^ CD24^high^CD38^high^ Bregs have previously been described in several autoimmune diseases. Patients with SLE and RA had similar peripheral blood levels of Bregs compared with healthy controls, and Bregs from SLE lacked the suppressive capacity^11^,^15^. Bregs in patients with pemphigus were elevated but with defective regulatory function on Th1 cells^16^. In patients with systemic sclerosis (SSc), Bregs levels were reported to be decreased almost by half compared to those in healthy individuals^19^. These findings suggest that the amount and function of Bregs may vary in the pathogenesis of different autoimmune diseases. This is the first report to discuss Bregs levels in DM, showing that Bregs levels are remarkably reduced in DM patients. The mean frequency of Bregs in DM patients was less than a fifth of that in healthy controls (0.713 ± 0.275% vs 4.196 ± 0.347%, p < 0.0001). And the prevalence of Bregs deficiency (defined as Bregs/B cells <0.50% in the current study) in DM patients was 73.3% (22/30). Such a low Bregs level in DM prompted us to find a reasonable explanation. However the factors involved in inducing Bregs have not yet been fully identified. Some factors and cytokines, including LPS, agonistic CD40 antibody, BAFF, binding immunoglobulin protein, IL-21 and IL-35^20^,^21^,^22^,^23^,^24^, have been reported to have the ability to induce the generation of Bregs, but their exact roles in Bregs production need to be verified in future studies.

It was important to make clearly that whether the frequency of CD19^+^ CD24^high^CD38^high^ Bregs in patients with DM could be affected by drug therapy. In this study, no difference between DM received drug therapy and treatment naïve DM patients was found regarding the frequencies of Bregs populations (p > 0.05). This result suggested that drugs might have no direct impact on the level of Bregs.

We further demonstrated that the frequency of IL10^+^ B cells was also significantly lower in DM patients than in healthy controls (1.139 ± 0.156% vs 2.372 ± 0.205%, *p* < 0.001), which is consistent with previous reports describing a similar trend in the CD19^+^ CD24^high^CD38^high^ Bregs and IL-10^+^ B cells of patients with other diseases^25^,^26^.

Several pieces of evidence indicate that CD19 molecules are important for Breg proliferation and survival. CD19-deficient (CD19−/−) mice is essentially devoid of regulatory B10 cells, which leads to exacerbated inflammation and disease symptoms during contact hypersensitivity and in the experimental autoimmune encephalomyelitis model of multiple sclerosis^27^. In the current study, the CD19 expression of B cells in patients with DM patients and healthy individuals were assessed and found to be lower in DM patients than in healthy individuals. It has been shown that Treg cells are not stable, can down-regulate the expression of FoxP3, and evolve into pathogenic Th1 cells or Th17 cells, depending on environmental factors such as inflammation^28^,^29^,^30^. Therefore, it is possible that Bregs are also an unstable subset, and that the reduced CD19 expression on B cells may be associated with decreased conversion of progenitor B cells into Bregs in DM patients.

Previous studies mainly focused on phenotypic characterization and functional analysis of circulating Bregs, with a few reports regarding the possible correlation between Breg deficiency and clinical features. We conducted a series of statistical analyses to unravel the relationships between CD19^+^ CD24^high^CD38^high^ Bregs levels and clinical/laboratory parameters. One interesting finding was the significantly lower percentage of CD19^+^ CD24^high^CD38^high^ Bregs in MSA-positive DM patients (*p* = 0.036). In addition, the Bregs levels of ANA-positive patients also displayed a trend toward being lower than those of ANA-negative patients. These results indicate that CD19^+^ CD24^high^CD38^high^ Bregs levels may have influence on the production of autoantibodies in patients with DM. The precise mechanism of how Bregs contribute to B cell hyperactivity and autoantibody production remains unclear, but there is a hypothesis that Bregs, CD4^+^ CD25^+^ Tregs, CD8^+^ Tregs, and other Treg subsets compose a regulation network and collectively regulate antibody production^30^.

Another important finding of this study is that DM patients with ILD often had lower Bregs levels than patients without ILD, which is consistent with the finding of a recent research showing that systemic sclerosis patients with decreased Breg levels often had ILD^19^. At present, there is no standard procedure to evaluate the severity of ILD, so we did not perform the correlation analysis between Bregs levels and severity of ILD. But lower levels of Bregs in DM patients with positive anti-MDA5 antibodies implied a possible correlation between Bregs levels and severity of ILD to a certain extent, for anti-MDA5 antibody was frequently detectable in amyopathic DM associated with rapid progressive ILD^31^. Since ILD can also be observed in other autoimmune diseases, we further performed the same analysis on diseased controls and found that all the four RA patients with ILD also showed remarkably decreased Breg levels (0.08%, 0.50%, 0.13% and 0.20%, respectively). But the mean percentage of Bregs in RA did not statistically differ from healthy individuals (3.450 ± 0.613% vs 4.196 ± 0.347%, p > 0.05).

Regarding the association between Bregs and DM disease activity, we performed a follow-up study that analyzed seven patients with DM before and after treatment. The Breg levels in these seven DM patients were found to be significantly higher after treatment than before (*p* = 0.022). However, when we examined if there was a correlation between change in MYOACT score (ΔMYOACT) and change in Breg/B cells (ΔBregs), no correlation was found (r = −0.208, *p* > 0.05) (Fig. 6C). One shortcoming of this follow-up study is the small number of subjects. It is certainly worth carrying out a larger size, longitudinal observational study to confirm the findings and determine how disease remission and flare-ups influence the Breg population.

Releasing IL-10 is the chief mechanism of Breg immunosuppressive activity, leading to the suppression of Th1 and Th17 responses, and down-regulating the production of proinflammatory cytokines, such as IFN-γ, IL-17 and TNF-α, from those effector cells^7^. Thus, in the present study, levels of IFN-γ, IL-17, TNF-α as well as of IL-10 were quantified. However, no notable relationship was observed between the percentages of CD19^+^ CD24^high^CD38^high^ Bregs and levels of these serum cytokines. TNF-α, IFN-γ and IL-17 levels were significantly higher in patients with DM than in healthy controls (*p* < 0.05). And elevated levels of these proinflammatory cytokines could be considered as a possible consequence of Breg reduction, which is also associated with Th1 and Th17 increase. However, we were surprised to find equivalent levels of IL-10 in serum samples from DM patients as with those from healthy individuals (*p* > 0.05). Given that regulatory cells constitute an important source of IL-10, we thought that serum IL-10 levels in DM patients might be significantly lower than those in healthy controls, considering that Bregs are remarkably reduced in DM patients. However, no difference was observed between the IL-10 levels of the two groups. This finding raises the intriguing possibility that there may be a significant non-Breg-driven IL-10 production response *in vivo* and that IL-10 can be produced from other cells during the complex inflammatory process. In fact, another high IL-10-expressing Breg subtype, namely CD25^high^FoxP3^high^ Bregs, has been described in SLE, the increase of this Breg subtype is considered to be one source of increased serum IL-10 in SLE patients^32^.

In summary, this is the first study to show a general and serious depletion of CD19^+^ CD24^high^CD38^high^ Bregs in patients with DM. The abnormally low quantity of Bregs in DM patients and the significant correlations between Breg levels and MSA and ILD involvement suggest that Breg deficiency is an immunopathogenic feature of DM and is part of the pathomechanism of this autoimmune disease. Our preliminary results may be an additional piece to help solve the puzzle of DM pathophysiology and provide insights into the development of new potential intervention targets for suppressing inflammation. However, the number of subjects is small in our study, so that the statistical differences we found in stratification analyses only suggest a trend of difference between sub-groups. A larger scale of study is needed to verify the reliability of these results. In addition, this preliminary study did not perform function verification due to the difficulty of gathering a sufficient number of Bregs in Breg-deficient DM patients. It will be interesting to study whether the Bregs in patients with DM display normal immunoregulatory activity; such a study could also help define the precise role of Bregs in the pathogenesis of this inflammatory autoimmune condition in future studies.

## Materials and Methods

### Patients

A total of 30 DM patients from China-Japan Friendship Hospital from June 2015 to October 2015 were enrolled in this study. A clinical diagnosis of DM was based on the classification criteria established by Bohan and Peter^33^. For comparison, 37 subjects with other autoimmune diseases were studied as well, including 15 cases of SLE, 15 cases of RA and 7 cases of pSS. Twenty-three age and sex matched healthy individuals (HC) were also included as normal controls. The demographic data of these patients are shown in Table 1. And all of the data was collected at the time of blood sampling. Peripheral blood samples for immunophenotyping were collected in heparin sodium anticoagulant and stored at room temperature until ready for staining and lysing. Sera were stored at −80 °C until analysis. This study was approved by the Ethics Committee of China-Japan Friendship Hospital and conducted in accordance with the Declaration of Helsinki guidelines. Informed consent was obtained from all patients for their participation in this study.

### Clinical assessment

The physical examinations and routine laboratory investigations were conducted for all patients at the time of blood sampling. Disease activity was assessed by using the 2005 myositis disease activity assessment tool (MDAAT) established by the International Myositis Assessment and Clinical Studies Group. The disease activity evaluation was also done at the time of blood sampling. Clinical diagnosis of ILD was based on impaired lung function and typical high-resolution computed tomography (HRCT) features (widespread ground-glass attenuation, intralobular lines/irregular interlobular septal thickening and honeycombing)^34^.

### Phenotypic measures of CD19^+^ CD24^high^CD38^high^ Bregs

The following anti-human monoclonal antibodies (MoAbs) were used for Bregs detection: allophycocyanin(APC)-labeled monoclonal anti-CD19; phycoerythrin(PE)-labeled monoclonal anti-CD24; fluorescein isothiocyanate (FITC)-labeled monoclonal anti-CD38; and isotype-matched and fluorochrome-matched control antibodies. All of the antibodies were purchased from Becton Dickinson Pharmingen (San Diego, California, USA).

Peripheral blood samples for immunophenotyping were collected in heparin sodium anticoagulant, and store anticoagulated blood at room temperature until ready for staining and lysing. To characterize the CD19^+^ CD24^high^CD38^high^ lymphocytes, 100 μL of whole blood cells were incubated with Human BD Fc Block (BD Biosciences)(2.5 μg, 10 minutes at room temperature) and then stained with anti-CD19 APC (50 μg/mL, 5 μL), anti-CD24 PE(12.5 μg/mL, 20 μL), and anti-CD38 FITC(6 μg/mL, 20 μL) as well as with their respective isotype control antibodies for 20 minutes in the dark at room temperature. Next, 2 mL of 1X FACS lysing solution (BD Bioscience) were added. The mix was vortexed gently and then incubated for 10 minutes in the dark at room temperature to hemolyze the red blood cells. The leukocytes were centrifuged and washed in PBS, and then suspended in 500 μL of PBS for flow cytometry analysis. The samples were analyzed on a FACSC JAZZ (BD Bioscience, San Jose, CA, USA) using FACS software (BD Bioscience). The gate strategy for Breg cell quantification was based on CD19, CD24 and CD38 expression, as shown in Fig. 1. The lymphocyte population was gated according to forward- and side-scattered properties, doublets were removed according to the trigger pulse width and CD19^+^ B cells were gated using anti-CD19 APC antibodies. Isotype control antibodies were used to separate positive and negative cells. According to the intensity of CD24 and CD38 expression, CD19^+^ B cells were subdivided into four populations: CD19^+^ CD24^high^CD38^high^ (Bregs), CD19^+^ CD24^intermediate^CD38^intermediate^ (primarily mature B cells), CD19^+^ CD24^high^CD38^−^ (primarily memory B cells), and CD19^+^ CD24^low^CD38^high^ (not clearly defined)^17^,^18^.

### Intracellular staining for IL-10

For analysis of the intracellular IL-10 produced by Bregs, heparinized blood samples were stimulated with Leukocyte Activation Cocktail (BD Biosciences), which is a polyclonal cell activation mixture containing the phorbol ester PMA (Phorbol 12-Myristate 13-Acetate); a calcium ionophore (Ionomycin); and the protein transport inhibitor BD GolgiPlug™ (Brefeldin A). Briefly, added 2 μL of cocktail directly for every 100 μL of heparinized blood samples and mix thoroughly. Then incubated for 6 hours in a 37 °C humidified CO_2_ incubator. After the activation step, activated cells were harvested and washed with FACS Staining Buffer (BD Biosciences), stained for surface markers and then washed, fixed, and permeabilized using a Cytofix/Cytoperm kit (BD Biosciences). For intracellular staining, cells were further stained with PE-labeled monoclonal anti–IL-10 from Becton Dickinson for 20 minutes in the dark at room temperature. Flow cytometry analysis was performed by FACS JAZZ.

### Serum cytokine analysis

Cytometric bead array (CBA) was used to quantify multiple cytokines simultaneously. In this study, Interleukin-10 (IL-10), Tumor Necrosis Factor (TNF), Interferon-γ (IFN-γ), and IL-17 protein levels were measured according to the manufacturer’s instructions. The samples were analyzed on a FACSCalibur system (BD Bioscience, San Jose, CA, USA) using BD CellQuest software (BD Bioscience).

### Statistical analysis

All analyses were performed using SPSS 13.0 for Windows and GraphPad Prism version 5.0 (GraphPad Software, Inc., San Diego, CA, USA). For continuous variables, results are expressed as mean ± SD; differences between two groups were analyzed by using unpaired *t* tests. For variables that are not normally distributed, results are expressed as the median and range; differences between groups were assessed using the Mann-Whitney *U* test. Correlations were determined by using Spearman’s rank correlation coefficients. P-values less than 0.05 were considered significant.

## Additional Information

**How to cite this article**: Li, W. *et al.* Significant decrease in peripheral regulatory B cells is an immunopathogenic feature of dermatomyositis. *Sci. Rep.* **6**, 27479; doi: 10.1038/srep27479 (2016).

## Supplementary Material

Supplementary Information

## Figures and Tables

**Table 1:** Demographic and clinical features of DM patients and controls.

		DM patients	Healthy controls	Diseased controls
Number		30	23	37^a^
Age (years)		47.3 ± 15.6	46.6 ± 13.1	50.8 ± 13.6
Sex (male: female)		9/21	7/16	10/24
Disease duration (months)		6 (0–132)	NA	NA
Major clinical features	*Skin rash*	100% (30/30)	NA	NA
	*Interstitial lung disease*	53% (16/30)	NA	11% (4/37)
	*Erythema*	90% (27/30)	NA	NA
	*Dysphagia*	27% (8/30)	NA	NA
	*Amyosthenia*	63% (19/30)	NA	NA
MSA positive		63% (19/30)	NA	NA
MYOACT score (mean ± SD)		4.2 ± 2.7	NA	NA
Patients received treatment		23	0	37
Treatment strategies	*NSAIDs*	0%	0%	100%
	*Glucocorticoids*	100% (23/23)	0%	73%^b^
	*DMARDs*	48% (11/23)	0%	100%
	*Biologics*	0%	0%	16%^c^

MSA: myositis specific autoantibodies in patients with DM, including anti-aminoacyl-tRNA synthetase antibodies, anti-Mi2, anti-SAE, anti-MDA5, anti-NXP2, or anti-TIF1-γ; NSAIDs: non-steroidal anti-inflammatory drugs; DMARDs: disease-modifying anti-rheumatic drugs. NA; not applicable.

^a^Diseased control group included 15 cases of SLE, 15 cases of RA and 7 cases of pSS; ^b^Of the 37 diseased controls, a total of 15 SLE patients, 10 RA patients and 2 pSS patients were treated with glucocorticoids; ^c^Of the 37 diseased controls, 6 RA patients were treated with biologics.

**Table 2:** Concentrations of serum cytokines in DM patients and in healthy controls.

Variable (pg/mL)	DM	Healthy controls	Statistical significance (*p* value)
IL-10 (median and range)	3.6 (2.4–24.7)	3.1 (2.1–8.9)	*p* > 0.05
TNF (median and range)	3.3 (2.9–142.9)	2.8 (1.9-4.9)	*P* = 0.018^a^
IFN-γ (mean ± SD)	9.54 ± 1.09	6.15 ± 0.37	*P* = 0.006^a^
IL-17 (mean ± SD)	23.91 ± 2.45	17.14 ± 1.36	*P* = 0.020^a^

^a^Difference between two groups is statistically significant.
