# Supplementary material for: Significant decrease in peripheral regulatory B cells is an immunopathogenic feature of dermatomyositis
Source: Sci Rep. 2016 Jun 7;6:27479. doi: 10.1038/srep27479 (PMC4895339; doi:10.1038/srep27479)
Supplement: Supplementary Information [file srep27479-s1.doc]

**Significant decrease in peripheral regulatory B cells is an immunopathogenic feature of dermatomyositis**

Wenli Li^1^, Xiaolan Tian^1^, Xin Lu^1^, Qinglin Peng^1^, Xiaoming Shu^1^, Hanbo Yang^1^, Yuanli Li^1^, Yan Wang^1^, Xuezhi Zhang^1^, Qingyan Liu, Guochun Wang^*^

^1^ Department of Rheumatology, China-Japan Friendship Hospital, Beijing, People’s Republic of China

**Supplementary Table**

Supplementary Table S1. Characteristics of patients with DM enrolled in the study

| No. | Age | Sex | Cinical Features  (Erythema/muscle weakness/ILD/dysphagia) | Serological Features | | MYOACT global disease activity score | Treatment |
| --- | --- | --- | --- | --- | --- | --- | --- |
|  |  |  |  | ANA | Myositis Abs |  |  |
| 1 | 34 | F | +/+/+/- | + | +: anti-EJ | 5.5 | - |
| 2 | 27 | M | +/+/-/+ | - | +: anti-NXP2 | 8.5 | - |
| 3 | 50 | M | +/-/+/- | - | +: anti-MDA5 | 3.5 | high CS, MMF |
| 4 | 46 | M | +/+/-/- | + | - | 4.8 | medium CS |
| 5 | 45 | M | +/+/+/+ | - | +: anti-MDA5, anti-PM-Scl-75 | 9 | high CS |
| 6 | 63 | F | +/+/+/+ | + | - | 8 | high CS |
| 7 | 17 | F | +/-/-/- | + | +: anti-Mi-2α | 2.5 | - |
| 8 | 64 | F | +/+/-/+ | + | +: anti-Mi-2α, anti-Mi-2β | 5 | high CS |
| 9 | 62 | F | +/-/+/- | - | +: anti-MDA5 | 5 | medium CS |
| 10 | 49 | M | +/-/+/+ | + | - | 0.5 | medium CS, MMF |
| 11 | 47 | F | +/+/-/- | + | +: anti-Mi-2α, anti-Mi-2β | 4 | high CS |
| 12 | 44 | F | +/-/-/- | + | - | 0.5 | low CS, MTX |
| 13 | 76 | M | +/-/+/- | - | +: anti-MDA5 | 7 | high CS |
| 14 | 35 | F | +/+/-/- | + | +: anti-Mi-2α | 3 | low CS |
| 15 | 53 | F | +/+/+/+ | - | +: anti-MDA5 | 9.5 | - |
| 16 | 61 | F | +/+/-/- | - | +: anti-TIF1γ, anti-Mi-2β | 3 | medium CS, MMF |
| 17 | 67 | F | +/+/-/- | - | +: anti-TIF1γ | 6 | - |
| 18 | 76 | F | +/+/+/- | + | +: anti-TIF1γ | 7 | - |
| 19 | 34 | F | +/-/-/- | - | - | 1 | medium CS, HCQ |
| 20 | 47 | F | +/-/-/- | - | - | 0 | low CS |
| 21 | 48 | F | +/+/+/- | - | - | 3 | high CS, AZA, HCQ |
| 22 | 27 | M | +/-/-/- | - | - | 3 | high CS, HCQ |
| 23 | 18 | M | +/-/-/- | - | - | 0 | low CS, HCQ, MTX |
| 24 | 64 | F | -/+/+/- | + | +: anti-MDA5 | 5 | high CS |
| 25 | 49 | F | +/+/+/- | + | +: anti-MDA5 | 5.5 | medium CS |
| 26 | 45 | F | +/+/+/+ | + | +: anti-SRP | 5 | high CS |
| 27 | 36 | F | +/+/+/+ | + | +: anti-EJ | 4 | high CS, AZA |
| 28 | 26 | F | +/+/-/- | - | - | 3.5 | - |
| 29 | 54 | F | -/+/+/- | + | - | 0.3 | medium CS, CPM |
| 30 | 54 | M | -/-/+/- | + | +: anti-PL-7 | 2 | high CS, MMF |

ILD: interstitial lung disease, EJ： glycyl-tRNA synthetase, NXP2: MJ-p140-MU 140kD protein, MDA5: melanoma differentiation-associated gene 5, Mi-2α: chromodomain-helicase-DNA-binding protein 3, Mi-2β: chromodomain-helicase-DNA-binding protein 4, TIF1γ: transcriptional intermediary factor 1-gamma, SRP: signal recognition particle, PL-7: threonyl-tRNA synthetase. CS: corticosteroids(low: ≤15 mg/day, medium: 15-30 mg/day, high: >30 mg/day), MMF: mycophenolate mofetil, MTX: methotrexate, HCQ: hydroxychloroquine, AZA: azathioprine, CPM: cyclophosphamide.
